# Bridging the Gap: Advances and Challenges in Heart Regeneration from In Vitro to In Vivo Applications

**DOI:** 10.3390/bioengineering11100954

**Published:** 2024-09-24

**Authors:** Tatsuya Watanabe, Naoyuki Hatayama, Marissa Guo, Satoshi Yuhara, Toshiharu Shinoka

**Affiliations:** 1 Center for Regenerative Medicine, The Abigail Wexner Research Institute, Nationwide Children’s Hospital, Columbus, OH 43205, USA; tatsuya.watanabe@nationwidechikdrens.org (T.W.); marissa.guo1@nationwidechildrens.org (M.G.); satoshi.yuhara@nationwidechildrens.org (S.Y.); 2 Department of Anatomy, Aichi Medical University, Nagakute 480-1195, Japan; nhatayama416@gmail.com; 3 Department of Surgery, Ohio State University, Columbus, OH 43210, USA; 4 Department of Cardiothoracic Surgery, The Heart Center, Nationwide Children’s Hospital, Columbus, OH 43205, USA

**Keywords:** cardiac regeneration, vascularization, heart tissue, 3D printing, transplantation, microvasculature, paracrine effect

## Abstract

Cardiovascular diseases, particularly ischemic heart disease, area leading cause of morbidity and mortality worldwide. Myocardial infarction (MI) results in extensive cardiomyocyte loss, inflammation, extracellular matrix (ECM) degradation, fibrosis, and ultimately, adverse ventricular remodeling associated with impaired heart function. While heart transplantation is the only definitive treatment for end-stage heart failure, donor organ scarcity necessitates the development of alternative therapies. In such cases, methods to promote endogenous tissue regeneration by stimulating growth factor secretion and vascular formation alone are insufficient. Techniques for the creation and transplantation of viable tissues are therefore highly sought after. Approaches to cardiac regeneration range from stem cell injections to epicardial patches and interposition grafts. While numerous preclinical trials have demonstrated the positive effects of tissue transplantation on vasculogenesis and functional recovery, long-term graft survival in large animal models is rare. Adequate vascularization is essential for the survival of transplanted tissues, yet pre-formed microvasculature often fails to achieve sufficient engraftment. Recent studies report success in enhancing cell survival rates in vitro via tissue perfusion. However, the transition of these techniques to in vivo models remains challenging, especially in large animals. This review aims to highlight the evolution of cardiac patch and stem cell therapies for the treatment of cardiovascular disease, identify discrepancies between in vitro and in vivo studies, and discuss critical factors for establishing effective myocardial tissue regeneration in vivo.

## 1. Introduction

Cardiovascular diseases are a major cause of morbidity and mortality worldwide, accounting for approximately 31% of all global deaths. Among these, heart failure resulting from ischemic heart disease has become increasingly prevalent, especially among an expanding aging population where risk factors such as hypertension, diabetes, and obesity are on the rise. Following myocardial infarction (MI), adverse cardiac remodeling secondary to the massive loss of cardiomyocytes, exuberant inflammatory response, degradation of the extracellular matrix (ECM), and tissue fibrosis lead to functional impairment [1]. While heart transplantation represents the only curative treatment option available for end-stage heart failure, the scarcity of donor organs necessitates the development of alternative therapies [2,3]. Although recent advancements in ventricular assist device technology have been remarkable, risks such as infection, bleeding, thromboembolism, and device thrombosis have remained significant issues with mechanical heart pumps.

Myocardial regeneration is challenging due to the limited proliferative capacity of cardiomyocytes [4]. The rate of turnover has been estimated to be as low as 0.45–1% per year, which is negligible in comparison to the loss of tissue resulting from MI [5]. In cases of heart failure and low cardiac function, methods to promote endogenous tissue regeneration by inducing vascular formation and increasing growth factors alone are insufficient for cardiac tissue regeneration [6]. Hence, the development of techniques for transplanting viable new tissue akin to partial grafts is highly sought after [7]. Research aimed at myocardial regeneration has been extensive, with preclinical studies focusing mostly on models of acute MI and clinical trials most often involving patients with chronic cardiomyopathy [8,9,10]. Several approaches to tissue transplantation, including epicardial patches, interposition grafts, and stem cell injections, have been investigated [11,12,13,14].

The establishment of a well-perfused vascular network is fundamental to the development of functional cardiac tissue, largely due to the significant metabolic demands of the heart. Angiogenesis plays a pivotal role in preserving tissue homeostasis within three-dimensional (3D) tissue constructs and ensuring the survival and functionality of cells by providing an adequate supply of oxygen and nutrients [15]. Blood vessels may be incorporated into engineered tissue using cell-based therapies and the administration of paracrine factors to induce vascular growth, or integrated into biomaterials created with tissue engineering techniques [16]. Stem cell-based therapies capitalize on the ability of stem cells, particularly mesenchymal stem cells (MSCs), to differentiate into multiple cell types and secrete proangiogenic factors that encourage neovascularization. Growth factors, such as vascular endothelial growth factor (VEGF) and fibroblast growth factor 2 (FGF-2), have also been studied extensively in cardiac regeneration for their proangiogenic properties. These growth factors can be targeted to the damaged heart tissue to stimulate the formation of new blood vessels, thereby enhancing tissue regeneration [17].

Overall, the most effective treatment modality may vary depending on the chronicity of the disease [18]. Therapies that reduce the area of infarct by promoting vascularization and stimulating endogenous repair mechanisms may be the most effective in cases of acute MI [19]. In contrast, patients with chronic heart failure or non-ischemic cardiomyopathy may require direct myocardial tissue transplantation, as paracrine effects alone offer limited therapeutic benefits in the setting of long-term ventricular remodeling and fibrosis [9,20,21].

Many preclinical trials have demonstrated the positive effects of tissue transplantation on vasculogenesis and angiogenesis. Nevertheless, the successful transplantation of established tissue is rarely reported in large animal experiments [22]. While adequate vascularization is essential for the survival of transplanted tissues, even tissues with pre-formed microvasculature have not achieved sufficient engraftment [23,24]. To address this, recent studies have reported increasing success in enhancing cell survival rates in vitro by enabling tissue perfusion. However, the transition to in vivo experiments remains a significant challenge, with promising results yet to be reported in large animal models.

The aim of this review is to highlight the evolution of cardiac patch and stem cell therapies for in vivo myocardial regeneration, summarizing the transitions from in vitro research. Additionally, we will identify the discrepancies between in vitro and in vivo studies, explore the challenges of applying in vitro techniques to in vivo models, and discuss critical factors for establishing effective cardiac tissue regeneration in vivo.

## 2. In Vitro Trails

### 2.1. In Vitro Studies

While in vivo studies on cardiac tissue transplantation often involve the addition of endothelial cells (ECs) as a method for pre-forming microvasculature or inducing in situ vascular formation through paracrine effects, an effective strategy for long-term tissue survival has not yet been established. Recent in vitro trials have focused on engineering tissues with established feeding arteries and drainage veins. These techniques have been used to create all types of artificial tissues in addition to cardiac tissue. For functional myocardial implants in particular, the generated tissue needs to be sufficiently thick, and adequate vascularization is essential to achieve a certain tissue thickness. The following sections will review the evolution and latest in vitro attempts to improve tissue engraftment and survival, as well as discuss the factors that could hinder the transition from in vitro to in vivo testing.

### 2.2. Securing Cellular Materials

It is estimated that in humans, adult-sized organs possess between 10 and 300 billion cells, with a cell density of approximately 100 million cells per milliliter [25,26,27]. In the human heart, it is estimated that there are 2 to 3 billion cardiomyocytes [28,29]. In reality, the heart is composed of at least 11 major cell types, including fibroblasts, ECs, smooth muscle cells (SMCs), immune cells, and several other subtypes, in addition to cardiomyocytes [25,30,31]. One of the challenges to recreating tissues and organs with the appropriate cellular density is a shortage of cellular materials [32]. The development of scalable cell production methods is essential to the ongoing progress of organ engineering. Induced pluripotent stem cells (iPSCs) avoid the ethical dilemmas associated with embryonic stem cells (ESCs) and provide a versatile source of pluripotent cells [33]. Moreover, iPSCs generated from individual cells have the potential to differentiate into multiple cell types, including cardiomyocytes, ECs, and SMCs, making them a promising candidate for cardiac tissue engineering [34,35,36,37]. Protocols to stimulate cardiac differentiation from iPSCs have been established, but their yield often depends on the cell line, and the production efficiency of human iPSC (hiPSC)-CMs is often low [38]. Additionally, hiPSC-CMs exhibit a fetal phenotype and gradually lose their proliferative capacity over time [39]. Therefore, having a substantial number of hiPSCs is a prerequisite for obtaining hiPSC-CMs on a large scale.

### 2.3. Static Culture

Planar monolayer culture has been the conventional method for culturing undifferentiated hiPSCs [40,41]. This standard culture technique has the advantage of maintaining the cells’ pluripotent status and high differentiation potential. Furthermore, two-dimensional (2D) monolayer culture is advantageous for cardiac differentiation because all cells are uniformly exposed to the growth factors, small molecules, and paracrine factors contained within the medium [36,38]. However, the main drawback of 2D culture systems is the limited surface area and therefore, low cell yield. Obtaining a sufficient number of cells is critical to the generation of human-scale tissues and organs, and existing planar culture requires a large number of culture dishes and a significant amount of culture medium, leading to substantial increases in culture costs and labor for cell culture technicians. Typically, 2D culture systems need to “scale out” to obtain sufficient cell quantities [42]. Standard flask-based culture methods also consume a lot of time and resources, and lack the ability to produce physiologically appropriate numbers of cells while maintaining proliferative capacity and preserving phenotype [43]. The use of new technologies to enable the large-scale production of clinically suitable cell sources is needed to overcome these hurdles.

### 2.4. Flow Culture

One of the methods to enhance culture efficiency involves creating a constant flow through the culture medium, ensuring sufficient oxygen supply and uniform distribution of nutrients. Traditional methods include rotational culture, where a cylindrical culture dish is rotated throughout the cultivation process. This technique allows cells to adhere to the entire circumference of the culture vessel, enabling cultivation with less medium compared to static monolayer culture, and facilitates smoother gas and metabolite exchange. The most basic method involves attaching cells to the walls of a tilted test-tube-shaped culture vessel inclined at 5 degrees, which is then rotated. This method yields a high cell harvest, and bottle-shaped culture vessels may be used as a large-scale culture apparatus. Additional techniques to perfuse the culture medium have also been devised [44,45,46]. Convection transport improves control over oxygen, nutrient, and metabolite levels, enabling the production of uniform artificial cardiac tissue. However, many of these perfusion cultures are intended for use as in vitro models in developmental biology, safety pharmacology, and drug discovery. Various designs have also been aimed at recreating cardiac-specific biomimetic environments by providing biochemical [47], mechanical [48,49,50], or electrical stimulation [51,52,53], and enhancing nutrient transport [44,45,46]. For example, Roberta et al. designed a bioreactor culture chamber that generates a 3D cardiac structure with bidirectional stromal perfusion and biomimetic electrical stimulation, allowing for direct optical monitoring of cells and contractility testing [54]. The combination of perfusion and electrical stimulation has been shown to promote tissue maturation, resulting in improved contractile force, enhanced beating characteristics, and increased expression levels of cardiac proteins.

### 2.5. Non-Gravity Culture

In suspension culture, agitation of the medium causes cells to proliferate in suspension. Cell culture in suspension may be adapted to a larger scale by increasing the size of the culture vessel. Suspension culture can also be conducted using standard dishes, and various culture devices have been designed for this purpose. This culturing system is simple and has the advantage of growing large quantities of cells compared to monolayer culture performed at the same scale. Recently, bioreactors have been developed to optimize culture conditions and proliferation capacity. Various bioreactor systems, such as rotating bioreactors, stirred-tank bioreactors, and hollow-fiber bioreactors, are used to proliferate iPSCs exponentially into quantities of billions [55,56,57]. Variations in bioreactor design aim to maximize the surface area for metabolic processes and enable proper oxygenation and nutrient delivery [43]. The combination of newer cell proliferation technology and established protocols for efficient iPSC reprogramming opens the door to creating human-scale organ-specific tissues. Nevertheless, the increased number of cells produced by such systems creates a more complex microenvironment, which carries the risk of spontaneous differentiation and hinders proliferative and directed differentiation capabilities [58].

### 2.6. 3D Construction and Vascularization

Recent research in tissue engineering and regenerative medicine has focused on efforts to artificially construct functional 3D tissues. The potential applications of such technology include the creation of artificial organs using cells obtained from individual patients and the development of tissues for in vitro drug testing as an alternative to animal experiments. Maturation of this field is therefore eagerly awaited. In this regard, 3D bioprinting has emerged as one of the most promising engineering technologies for manufacturing tissues in vitro, paving the way for the generation of thick cell-laden structures. However, as the thickness of the tissue graft increases, new obstacles arise. Currently, the greatest challenge to growing tissue constructs of sufficient size is the issue of mass transfer limitations. Physiologically, most cells exist within 100 to 200 μm of the nearest capillary, which allows for the adequate diffusion of oxygen, nutrients, and waste products [59,60]. It may be extrapolated that when transplanting laboratory-grown tissues in vivo, only cells within 100 to 200 μm of the nearest capillary can survive due to this diffusion limitation [60]. Therefore, pre-formation of vascular structures within the tissue is critical before transplantation. Considerations when engineering vascularized tissue grafts include what cell types to incorporate, the diffusion rates of oxygen and nutrients, overall structural size, and the anticipated method of integration with the host vascular system. In the laboratory, this diffusion limitation is the main reason why constructing tissues thicker than a few hundred microns is currently not feasible. Gradients within the tissue constructs result from an unequal distribution of oxygen and nutrients, leading to spatial variations in cell number, viability, and phenotype [61].

### 2.7. Top-Down and Bottom-Up Approaches

The technical methods for constructing 3D tissues can be broadly classified into two types: top-down and bottom-up approaches [62]. The top-down approach involves constructing 3D tissues by seeding cells onto relatively large (millimeter-scale or greater) biodegradable scaffolds made of hydrogels or biocompatible polymers [63]. In contrast, the bottom-up approach entails preparing a large number of small (approximately 100 μm) tissue units and assembling them as building blocks to construct 3D tissues [64].

In bottom-up tissue engineering, the building blocks may be classified into three main types according to shape: planar (e.g., cell sheets), point (e.g., spheroids and cultured hydrogel blocks), and linear (e.g., cell-cultured hydrogel fibers) [65]. Traditionally, the approach to creating artificial tissues has been top-down, where cells and signaling molecules are introduced into 3D biomaterials that function as scaffolds to mimic the structure of the extracellular tissue environment. Recently, interest has shifted towards the bottom-up approach, where larger cellular structures, such as spheroids and cell sheets, are prepared by self-assembly and then used to construct larger and more complex structures [66]. Generally, 3D bioprinting can be considered a combination of top-down and bottom-up methods. Bio-inks composed of synthetic, natural, or mimetic polymers, cells, and various growth factors are deposited to create 3D structures that can structurally mimic tissues and organs, as well as replicate their physiological functions.

### 2.8. Spheroids

Spheroids are round cellular aggregates formed in suspension through intercellular adhesion where they are loosely in contact with each other. The use of cardiac spheroids may contribute to enhanced tissue generation, transplantation, and recovery following injury to the myocardium [67,68,69]. Spheroids can be generated using the aforementioned bioreactor. Additionally, simpler methods for culturing cells into spheroids involve the use of plates with U-shaped wells that have low cell adhesion. Some cells form spheroids simply when seeded onto non-adherent culture dishes [67,70,71]. In general, spheroids are obtained from one or more cell types that spontaneously form heterogeneously sized cell clusters [67,72]. The terms organoids and spheroids are often used interchangeably, but organoids are described as more complex 3D cell systems with structures closer to natural organs than spheroids [73,74].

Major challenges within the field of cardiac regeneration currently include tissue size restrictions, graft heterogeneity, cardiomyocyte immaturity, and lack of angiogenesis. Contemporary studies are aimed at addressing these issues. For example, the co-culture of hiPSC-CMs with fibroblasts has been shown to enhance the cardiac properties of transplanted tissues compared to 3D cultures containing hiPSC-CMs alone. The tri-culture approach of CMs, fibroblasts, and early vascular cells derived from hiPSCs has led to the generation of cellular aggregates with increased contraction rates, improved microvascular performance, enhanced spheroid formation, and more uniform distribution of CMs [75].

### 2.9. 3D Cell Blocks

The ability to create 3D cardiac structures has been demonstrated in several in vitro studies [76,77,78]. Typically, scaffolds, such as collagen, fibrin gel, or oriented fibers, are used to provide cells with a 3D structural environment. For instance, Ronaldson-Bouchard et al. reported the development of a functional 3D cardiac model using fibrin gel [79]. Although their model could partially replicate cardiac-specific functions, the cardiomyocytes within the gel did not fully recreate the high-density cellular state and cell–cell interactions present in native tissues. In addition, the surfaces of scaffold materials have been found to absorb hydrophilic or hydrophobic agents, making it difficult to evaluate accurate responses during drug testing [80].

Therefore, approaches in cardiac tissue engineering that do not use such scaffolds are gaining attention. Scaffold-free methods do not have the drawback of incorporating foreign materials and have been shown to produce tissue that is comparable to that generated by biomaterial-based methods. For instance, a 3D bioprinter can be used to place cardiac spheroids on a needle array, creating scaffold-free tubular cardiac structures [81]. This system also allows for contraction analysis by observing changes in the movement of the needle array tip. Nevertheless, this technique relies on the structural support of the needle array. In another method, 3D cardiac tissues with contractile characteristics and electrophysiological properties similar to native heart tissue have been made by seeding cardiac spheroids onto a metal net mold. The mold is eventually removed, resulting in a 3D construct without the need for support structures [12,82,83].

### 2.10. Vascular Network

**Figure 1:**
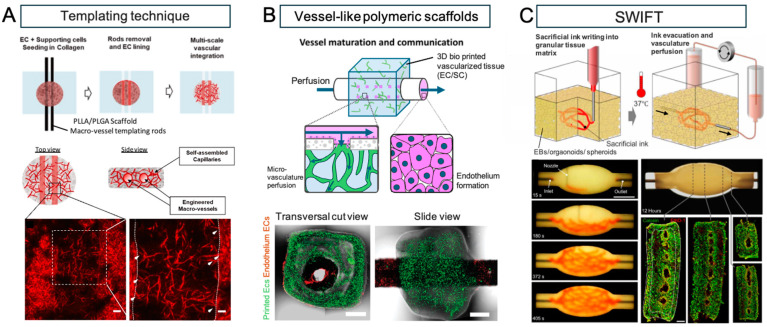
Perfusable vascular networks in 3D engineered tissue. (**A**) An approach by multi-scale vascular networks (MSVTs) comprised of patterned macro-channels integrated with self-assembled micro-vessels, within 3D engineered tissues. The engineered macro-vessels anastomose with the surrounding micro-vasculature can be observed in histological images (white arrows indicate micro-vessels sprouts; dashed lines indicate the macro-vessels borders). (white bar: 100 μm (left), 50 μm (right)). (**B**) Millimetric vessel-like scaffolds and 3D bioprinted vascularized tissues interconnect, creating fully engineered hierarchical vascular constructs for implantation. The spontaneously formed vessels in the hydrogel communicate with the endothelium through the scaffold fenestrations, enabling the microvasculature perfusion. Representative confocal images of assembled vascular constructs with endothelium ECs (dTomato-ECs, red) and printed microvascular ECs (ZsGreen-ECs, green) after one week in culture (white bar: 100 μm). (**C**) Sacrificial writing into functional tissue (SWIFT). Perfusable EB tissue fabricated by SWIFT. An image sequence showing the embedded 3D printing of a branched, hierarchical vascular network within a compacted EB-based tissue matrix connected to inlet and outlet tubes, seen entering the tissue from the left and right (white bar: 10 mm). Image of the perfusable tissue construct after 12 h of perfusion and fluorescent image of LIVE/DEAD (green/red) cell viability stains at various sections through the tissue (white bar: 10 mm). Reprinted from refs. [40,63,95] with Copyright permission.

The creation of angiogenic tissue has long been a goal of bioengineering, and various techniques have been developed to generate vascular tissue models in vitro [84,85,86]. Methods for constructing large blood vessels often rely on a top-down approach, which includes micro-molding with molds and needles, direct 3D printing of vascular channels into cell-laden biomaterials, or the use of multi-ink 3D printing that includes sacrificial biomaterials to form vascular lumens (Figure 1). However, due to limitations in manufacturing resolution, these methods cannot be used to generate microvasculature. Instead, microvasculature is often created through the self-organization of ECs into vascular networks known as plexuses. Many of the studies on EC self-organization are confined to small areas using microfluidic devices aimed at evaluating drug metabolism. Methods to create hierarchical vascular networks for transplantation have involved the implementation of large media channels running parallel to self-organized endothelial plexuses embedded within central hydrogels [87,88,89,90,91,92,93,94]. For instance, Figure 1.

Debbi et al. created a thick multiscale vascular network (MSVT) tissue by combining live cells, biological hydrogels, and biodegradable synthetic polymers. Macrovascular structures were manufactured by setting rods into collagen gel seeded with ECs and SMCs, which resulted in the creation of endothelialized vasculature after rod removal. Subsequent cultivation demonstrated the formation of anastomoses between these larger vessels and surrounding microvasculature [89]. Szklanny et al. bioprinted ECs and supporting cells into a microvascular network, then used collagen bio-ink to insert a polymer vessel within the center of the gel. The resulting artificial tissue was anastomosed to the rat femoral artery, with inward growth of vasculature from the host to the tissue graft [91]. 

Notably, a commonly cited issue with self-organized EC plexuses is that these vascular networks remain open at the gel boundaries.

The need to construct artificial tissues with vascular networks increases the complexity of this in vitro process, necessitating sophisticated 3D bioprinting technologies, advanced materials, and optimized biological protocols [96,97]. While several different printing methods fall under the bioprinting category, only a few have been effectively used to obtain vascularized structures. Extrusion bioprinting is the most cost-effective and versatile technique, allowing customization from an engineering perspective (e.g., multi-print heads, coaxial nozzles, microfluidic extrusion) [98,99,100], and showing great flexibility in material selection. Sacrificial inks are fundamental to the bioprinting of vascularized structures and must enable extrusion in air or within a support bath, later liquefying and washing away to leave hollow structures within another surrounding material [101]. Examples of sacrificial inks include Pluronic F-127 (PLU) [96,101,102,103], carbohydrate glass [104], gelatin [26,105,106], and agarose [107]. Kolesky et al. used PLU to create a series of vascular channels within a methacrylated gelatin (GelMA) chip. Human umbilical vein endothelial cells (HUVECs) were then seeded into these channels, resulting in perfusable vascular channels with an endothelial layer [103]. Additionally, methods such as freeform reversible embedding of suspended hydrogels (FRESH) can be used to accurately reproduce components of the human heart ranging from capillaries to the full organ [26,31].

In a study by Noor et al., cells were extracted from a patient’s omentum, reprogrammed to become pluripotent, differentiated into cardiomyocytes and ECs, and then encapsulated within hydrogels as personalized bio-inks [97]. Using gelatin as a sacrificial bio-ink, cardiac patches were printed with an extrusion-based 3D bioprinter. With an incubation temperature of 37 °C, the gelatin was liquefied and washed away, creating vascular lumens approximately 300 μm in diameter within the cardiac patch. As a proof of concept, a small-scale human heart measuring 20 mm in height and 14 mm in diameter with major blood vessels was created. This study garnered considerable attention; however, these tissues were not perfused for long periods, and cell viability and contractile function were not reported.

Other reports have described a novel technique for introducing perfusable vascular channels into tissue blocks using 3D bioprinting called sacrificial writing into functional tissue (SWIFT) [26]. In this method, hiPSC-derived organ building blocks (OBBs), such as spheroids or organoids, are combined with a tailored ECM solution to create a high-density living matrix in which a vascular network may be embedded using sacrificial ink. In one study, cardiomyocytes derived from embryoid bodies were mixed with an ECM solution of collagen I and Matrigel, as well as human neonatal dermal fibroblasts, to form a slurry, which was then centrifuged to create a cardiac tissue matrix with a cardiomyocyte density of 180 million cells per milliliter. This was then formed into disc-shaped molds approximately 6 mm in diameter, and after seven days of culture, the tissue structures were reported to beat spontaneously and in synchrony with rapidly propagating calcium waves. Vascular channels were embedded within these constructs using 3D bioprinting and perfused at 500 microliters per minute. Spontaneous beating was able to be maintained for eight days, and sarcomere remodeling was observed within the cardiac tissue. These results suggest that the creation of perfusable, organ-specific tissues of any volume and shape may be possible. However, issues such as low contractility due to insufficient cell maturation and microvascular network formation were cited.

While the fabrication of vascular networks using 3D printing methods appears to be an attractive technology in vitro, significant obstacles remain before it can be applied in an in vivo setting. Currently, there are no established methods to create small-diameter vasculature to connect with existing blood vessels in the host tissue. Moreover, it is unknown whether artificially constructed blood vessels or hydrogel scaffolds can withstand arterial pressures. Meanwhile, increasing the hydrogel’s strength in cardiac tissue constructs may reduce contractile function and cell viability. Additionally, the risk of thrombosis within the fabricated tissues is another concern. These represent a few of the considerations that need to be addressed before such advanced tissue engineering techniques may be translated into in vivo models.

## 3. In Vivo Trials

### 3.1. Preclinical Studies

Studies in cardiac regeneration conducted in vivo can be broadly categorized into cardiac patches and stem cell injections. Patch or sheet implants have undergone significant research due to their potential to provide structural support, promote vascularization, and deliver therapeutic cells or paracrine factors directly to the damaged myocardium. Cardiac patches may be divided into those without growth factors or cellular components, those with added growth factors or cellular components, and those with only cellular components. Cellular patches may include stem and ECs, which predominantly contribute to cardiac tissue repair through paracrine signaling, or cardiomyocytes for myocardial engraftment. Alternatively, single-cell suspensions or stem cell aggregates may be injected directly into the myocardium or infused through vascular routes. Stem cells administered in this method home to the injured myocardium and exert a cardioprotective effect by stimulating endogenous repair mechanisms. Several cell lines, including cardiac stem and progenitor cells, ESCs, iPSCs, and MSCs harvested from extracardiac tissues, such as bone marrow, adipose, and umbilical cord tissues, have been investigated as potential sources of cardiomyocytes on differentiation [108].

#### 3.1.1. Extracellular Matrix (ECM) Patches

ECM patches provide a scaffold for cellular attachment and growth. These patches are typically composed of natural or synthetic biomaterials, such as collagen, alginate, and decellularized tissues, that mimic the native cardiac ECM in an effort to create a conducive environment for cell infiltration and engraftment. A study by Ota et al. was performed to characterize the remodeling of an ECM-derived cardiac patch used as an interposition implant in the right ventricle of a porcine model [13]. They reported that the ECM scaffold was repopulated by α-smooth actin-positive cells 60 days after implantation, and the presence of these cells corresponded to areas within the patch that showed early signs of electrical conductivity. Sarig et al. conducted a study to evaluate the use of a porcine cardiac ECM patch in rat models of acute and chronic MI [109]. They found that the porcine cardiac ECM was rapidly vascularized, promoted constructive remodeling processes, and stimulated recruitment of cardiomyocyte progenitor cells. Nevertheless, the myocardial regeneration induced by simple patch transplantation is usually insufficient, and functional recovery is limited, especially in large animal models.

#### 3.1.2. Cell Sheets and Cell Blocks

The anticipated effects produced by patches with cellular components vary depending on the type of cells being transplanted. While the benefits of stem cell transplantation result primarily from the protective effects stem cells exert on the endogenous cardiac tissue, the intended outcome of transplanting cardiomyocytes is donor cell engraftment and functional support of the host myocardium. However, several challenges have arisen to the successful engraftment and functional integration of cellular patches.

In a study by Matsuzaki et al., hiPSC-CMs were seeded onto a biodegradable polymer sheet, which was then implanted as an interposition graft in rat hearts [110]. Two months after transplantation, cardiomyocytes were not observed on the explanted patch, suggesting the possibility of cell wash-out or cell death. Simple seeding of cells onto a sheet makes it difficult to establish more than a monolayer of cardiomyocytes and limits the number of transplantable cells. Additionally, inadequate adhesion to the host tissue may contribute to poor survival. Advancements in cell sheet engineering technology, however, have allowed for the production of thicker, functional myocardial tissue through an approach that involves stacking layers of cell sheets. This is often done using temperature-responsive culture dishes to facilitate cell sheet detachment without enzymatic treatment, preserving cell–cell junctions and ECM components. Using a strategy to enhance cell survival, Sekine et al. developed cell sheets created from the co-culture of cardiomyocytes with ECs. They found that epicardial implantation of EC-positive cell sheets in infarcted rat hearts led to higher capillary density within the transplanted tissue and bridging of EC-derived vasculature with capillaries of the host myocardium. Moreover, rats that received EC-positive cell sheets exhibited improved recovery of cardiac function compared to those implanted with EC-negative sheets [111].

Kawamura et al. conducted a study investigating the safety and therapeutic potential of hiPSC-CM sheets using a porcine ischemic cardiomyopathy model. They found that epicardial implantation of hiPSC-CM sheets led to improved cardiac performance and attenuated left ventricular remodeling. However, despite these benefits to cardiac function, very few transplanted hiPSC-CMs survived long-term [8]. In a subsequent experiment, Kawamura et al. showed that the survival rate of transplanted cells improved when hiPSC-CM sheets were implanted with an omental flap as a result of increased microvasculature formation mediated by growth factors including vascular endothelial growth factor (VEGF), basic fibroblast growth factor (bFGF), and stromal cell-derived factor 1 (SDF-1) supplied by the omentum [112].

With the use of allogeneic cells, the host immune reaction becomes another critical factor affecting cell survival. Strategies to minimize the risk of host rejection are needed for iPSC-based regenerative therapies. Nevertheless, Kashiyama et al. found that the allotransplantation of iPSC-CM sheets in cynomolgus macaques resulted in similar improvements to cardiac function in both major histocompatibility complex (MHC)-matched and MHC-mismatched animals, although vascular formation and myocardial tissue perfusion were more enhanced in the MHC-matched group [23]. Nevertheless, in spite of these findings, donor cells were no longer detectable after six months in either group. These results suggest that even without an immunologic reaction, transplanted cells do not survive long-term.

#### 3.1.3. Three-Dimensional (3D) Bioprinted Cardiac Patches

Biomaterial-free cardiac patches may be created using contemporary 3D bioprinting technology. Using cellular spheroids containing cardiomyocytes, ECs, and fibroblasts, Yeung et al. developed 3D bioprinted cardiac patches, which they implanted into a rat model of MI [19]. Cardiac patch implantation was associated with significantly reduced scar tissue formation, increased angiogenesis, and enhanced cardiac function. The use of a multicellular approach to cardiac tissue regeneration was further supported by study by Kim et al. [113]. The incorporation of both hiPSC-derived ECs and genetically engineered MSCs secreting SDF-1α into their 3D cardiac patch resulted in augmented angiogenesis and vasculogenesis, leading to enhanced cardiac function in rats with ischemic cardiomyopathy. Finally, although Kawai et al. sought to apply their 3D bioprinted tissue graft for a different purpose, a recent study published by their group is worth highlighting given its implications for the current and future status of cardiac tissue engineering. In an effort to create a beating Fontan conduit, Kawai et al. created scaffold-free cardiac tissue grafts comprising various cell spheroids, including iPSC-CMs, human umbilical vein endothelial cells (HUVECs), and fibroblasts [114]. These grafts were implanted around the abdominal inferior vena cava (IVC) of mice, and examination one month after transplantation showed pulsatility, as well as neovascularization and clear striation, of the implanted myocardial tissue.

Koda et al. investigated the feasibility of implanting 3D spheroid-based patches in a large animal model using healthy swine. Their patch was composed of cardiomyocytes and embryonic fibroblast cells and reinforced with an ECM. The implanted patches improved regional tissue perfusion and exhibited electrical activity. Furthermore, histologic analyses confirmed the presence of premature cardiomyocytes and active vasculogenesis, suggesting the potential for functional myocardial regeneration [12].

Although the successful engraftment and survival of transplanted tissues have been reported, many of these experiments have been conducted in rodents, where the size of the transplanted tissue is very small. Moreover, rodents have been shown to have relatively few rejection reactions, as well as rapid vascularization of transplanted tissues, circumventing some of the challenges seen in large animal models [115]. In large animals, the beneficial effects associated with cardiac patch implantation are more often attributed to the release of paracrine factors rather than donor cell survival.

A major challenge to cardiac tissue transplantation in large animals is the need for the rapid vascularization and establishment of connections with host blood vessels to provide adequate perfusion to the transplanted tissue. Notably, in vivo strategies to augment vascular formation within the cardiac patch focus on the addition of ECs or stimulation of branching microvasculature from the host myocardium. However, there are concerns regarding whether or not sufficient blood flow for tissue survival can be achieved through microvasculature alone, especially for larger patches. To address this issue, Endo et al. established a method to induce the formation of feeding arteries and drainage veins for cardiac cell sheet in situ. Although this has not yet been applied to the heart, the ability to form larger feeding arteries may increase blood perfusion and improve the survival rate of transplanted cells [116].

#### 3.1.4. Patches with Growth Factor

The addition of paracrine factors to cardiac patches can significantly enhance their regenerative potential. Growth factors such as VEGF and bFGF promote angiogenesis and tissue repair. Zimmermann et al. demonstrated that incorporating VEGF into a cardiac patch composed of cardiomyocytes and ECs seeded onto a biodegradable scaffold resulted in the functional integration of transplanted tissue and improved cardiac function in rats after MI [50]. The VEGF-loaded patch enhanced vascularization, cell survival, and myocardial repair, demonstrating the synergistic effects of combining growth factors with cellular therapies. Shafiq et al. designed a polycaprolactone/collagen type 1-based cardiac patch capable of eluting neuropeptide substance P (SP) and insulin-like growth factor-1c (IGF-1c) to recruit stem cells and promote vascularization. The study demonstrated that the SP and IGF-1c/SP patches significantly improved heart function and attenuated adverse cardiac remodeling in murine models of acute MI. Both the SP and IGF-1c/SP patches promoted neovascularization, though the IGF-1c/SP patch-treated group showed the highest numbers of newly formed vessels [117].

**Figure 2:**
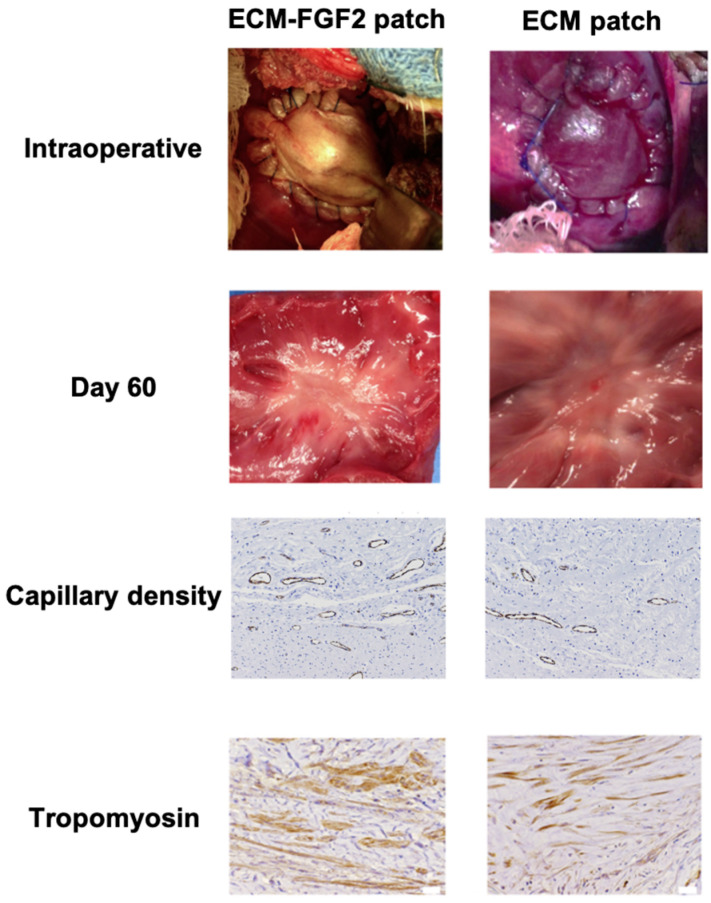
Effects of Growth Factor-Enhanced Patch. Compared to the ECM patch, the ECM patch supplemented with FGF2 (Fibroblast Growth Factor 2) has shown an increase in capillary density and a higher number of tropomyosin-positive cells. Adapted from ref. [14].

In a study by Tanaka et al., ECM patches loaded with bFGF were implanted as a right ventricle interposition tissue graft in swine. The authors reported that their patch facilitated the constructive repopulation of host cardiomyocytes, increased regional contractility and perfusion, and showed electrical activity [14] (Figure 2).

Cardiac patches loaded with growth factors may provide a similar paracrine effect to stem cells in the acute phase while avoiding the adverse effects associated with the administration of cells. However, the long-term release of growth factors will depend on the properties of the materials used, necessitating further research to identify the ideal materials for this purpose. Although there are no studies comparing the benefits of stem cell transplantation to treatment using growth factors only, this is an important area of future research.

#### 3.1.5. Injectable Stem Cell-Based Therapies

Unlike cell sheets and patches, which are implanted onto the heart en bloc, injectable therapies allow for the dispersion of stem cells within the host tissue. Several techniques have been established for the administration of single-cell suspensions or stem cell aggregates [118]. Intramyocardial injection through a transepicardial approach is the most direct method of delivery, circumventing the complex issue of stem cell homing. However, this procedure is the most invasive and requires entry into the chest through a thoracotomy. Transendocardial injections into the myocardium may be performed using a catheter-based approach, though these procedures require extensive imaging guidance to position the catheter at the desired site of injection. Intravenous infusion is the least invasive method of stem cell delivery, but this technique is reliant on physiological homing signals to the injured myocardium, which are present only after acute MI. Finally, stem cells may be administered through intracoronary arterial injection, which can allow for the selective infusion of cells through the coronary artery of interest. The major limitation to this technique is the inherent difficulty of delivering cells to areas with poor perfusion. Of note, an experiment by Hou et al. using radiolabeled cells showed that among intramyocardial, intracoronary, and retrograde transcoronary venous injections, the direct transepicardial approach yielded the highest rates of MSC retention [119].

In 2001, a pioneering study by Orlic et al. revealed that the intramyocardial injection of c-Kit+ bone marrow-derived mesenchymal stem cells (BM-MSCs) into infarcted mouse hearts led to the formation of new myocytes, ECs, and SMCs. Furthermore, mice that were treated with BM-MSCs exhibited improved left ventricular (LV) function and decreased infarct size compared to control mice [120]. Since then, countless preclinical studies in both small and large animal models have corroborated the beneficial effects of stem cell therapy on functional recovery, ventricular remodeling, and revascularization after cardiac injury. Funakoshi et al. observed that hiPSC-CMs injected into the infarcted hearts of immunodeficient mice grew substantially within the first month, gradually slowed in proliferation over the next two months, and then lost their proliferation capacity upon maturation thereafter [121]. Shiba et al. reported that allogeneic iPSC-CMs survived for at least 12 weeks and showed electrical coupling with host CMs when injected into the infarcted myocardium of immunosuppressed cynomolgus monkeys. Primates that received iPSC-CMs also exhibited improved contractile function at 4 and 12 weeks post-transplantation [122]. Nevertheless, multiple studies have reported that while they, too, observed improvements in cardiac function and remodeling associated with stem cell injection, these results occurred despite poor long-term donor cell engraftment [123,124,125].

The predominant challenge limiting the efficacy of injectable stem cell-based therapies is thought to be low retention and survival rates post-transplantation. Collantes et al. reported retention rates of only 13% and 17% soon after intramyocardial and intracoronary injections of CSCs in swine with chronic ischemic reperfusion injury. Moreover, they found that none of the cells that were delivered by intracoronary infusion were detectable on histological examination three days later [126]. Strategies that have been explored to increase the longevity of transplanted cells include the use of injectable biomaterials and the injection of multiple cell types. Injectable polymeric scaffolds can increase cell engraftment and survival by providing temporary biomechanical support, as well as enhancing angiogenesis [127]. Meanwhile, the combined delivery of multiple cell types may be advantageous as the actions of one cell type may augment the survival and function of others. A multicellular approach by Martinez et al. using human umbilical cord-derived MSC (UM-MSC) aggregates coated with HUVECs showed that the intramyocardial injection of these angiogenic spheroids produced improvements in cardiac function, tissue vascularization, and infarct size in rat models of MI [128].

The benefits to cardiac function and remodeling-associated stem cell transplantation were initially thought to be a result of myocardial tissue regeneration due to donor cell differentiation into new CMs. However, accumulating evidence has instead suggested that the cardioprotective effects resulting from stem cell-based therapies arise predominantly from the secretion of paracrine factors that stimulate endogenous repair mechanisms within the host myocardium. Gnecchi et al. found that functional recovery took place less than 72 h after the injection of BM-MSCs, which was too rapid of a response to be attributed to cardiac regeneration. Furthermore, they demonstrated that the injection of BM-MSC conditioned medium led to similar benefits to ventricular contractility and infarct size [129]. The paracrine hypothesis is now widely accepted by all researchers working in the field of cell therapy. Stem cells release a variety of cytokines and growth factors to recipient cells within the host tissues. These molecules interact in numerous pathways that promote angiogenesis and inhibit inflammation, fibrosis, and apoptosis [130,131]

### 3.2. Clinical Trials

Numerous early phase clinical trials have been conducted evaluating the safety, feasibility, and efficacy of stem cell-based therapies in patients with ischemic cardiomyopathy (Table 1 and Table 2). Intracoronary arterial infusion is the most clinically practiced method of stem cell delivery and can be performed during a percutaneous coronary intervention for acute MI. The results of such trials vary widely, with some studies reporting significant improvements in cardiac function associated with stem cell infusion while others found no differences in outcomes between treatment and control groups [95,118,132,133,134,135,136,137]. Published data on the effectiveness of intramyocardial stem cell injection in humans have also been inconsistent [138,139,140,141,142,143,144,145,146,147,148,149,150,151,152].

Other trials involve device-based interventions, such as CorMatrix^®^-ECM^®^ and VentriGel, for epicardial repair in post-MI patients. Clinical trials making use of cardiac patches are limited. Menasché et al. performed the first clinical study investigating the therapeutic potential of ESCs in the setting of ischemic cardiomyopathy. ESC-derived cardiovascular progenitor cells embedded in a fibrin patch were implanted onto the epicardium overlying the area of infarction in six patients with severe LV dysfunction who were concurrently undergoing CABG. The four patients who were assessed at the 1-year follow-up time point were reported to have symptomatic improvement, improved LV ejection fraction (LVEF), and enhanced wall motion of the patched segments. No tumors or arrhythmias were detected postoperatively. Nevertheless, the lack of a control group, small sample size, and simultaneous revascularization at the time of ESC patch implant make it impossible to draw any conclusions [153]. A larger study by Domae et al. examined the epicardial implantation of autologous skeletal muscle patches in 24 cases of dilated cardiomyopathy. Follow-up at six months showed that there were both responders, who showed improvement in symptoms and exercise tolerance, and non-responders, who showed little to no improvement. The beneficial effects of cardiac patch transplantation have been attributed to the induction of angiogenesis and cardiomyogenesis through paracrine mechanisms. The BioVAT-HF trial represents the only ongoing clinical trial evaluating the surgical implantation of tissue-engineered myocardium in patients with end-stage heart failure.

**Table 1:** Published results of human clinical trials with cardiac patches.

Reference	Patch Type	Target Disease	Implantation Method	Number of Patients	Follow-Up Duration	Patient Outcomes
[154]	Allogeneic human iPSC-derived cardiomyocyte patches	Ischemic cardiomyopathy	Thoracotomy, left ventricle epicardium	1	3 months	Improved clinical symptoms, no major adverse events, potential tolerance to exercise, no tumorigenesis detected
[20]	Autologous skeletal stem cell patch	Nonischemic dilated cardiomyopathy	Left thoracotomy, anterior and lateral left ventricle epicardium	24	6 months	Improvement in symptoms, exercise capacity, and cardiac performance in responders. Better actuarial survival rate
[9]	iPSC-derived cardiomyocyte patches	Heart failure	Thoracotomy, left ventricle epicardium	4	6 months	Improvement in cardiac function, safe transplantation, no severe adverse immune responses
[155]	Human ESC-derived cardiac progenitor cells in a fibrin scaffold	Severe heart failure	Thoracotomy	1	6 months	Improved functional status, no adverse events, reduction in LV end-diastolic and end-systolic volumes

**Table 2:** Human clinical trials for cardiac patches.

NCT Number	Study Title	Acronym	Study Status	Conditions	Interventions	Target Disease	Implantation Method	Primary Outcome Measures	Secondary Outcome Measures	Phases	Enrollment
NCT02887768	Epicardial Infarct Repair Using CorMatrix^®^-ECM^®^	EIR	COMPLETED	Acute Coronary Syndrome, Heart Failure	Epicardial Infarct Repair with CorMatrix^®^-ECM^®^	Heart Failure	Epicardial implantation	Incidence of serious adverse events	Number of patients in which the target myocardial thickness is maintained	EARLY_PHASE1	8
NCT02305602	A Study of VentriGelin Post-MI Patients	NaN	COMPLETED	Myocardial Infarction, Heart Failure, Left Ventricular Dysfunction	VentriGel	Post-ML	Intramyocardial injection	Incidence of serious adverse events	NaN	PHASE1	15
NCT02057900	Transplantation of Human Embryonic Stem Cell-derived Progenitors in Severe Heart Failure	ESCORT	COMPLETED	Ischemic Heart Disease	Human embryonic stem cell-derived cardiac progenitors	Severe Heart Failure	Epicardial implantation	Number and nature of adverse events, Evidence of functional improvement	Feasibility of patch’s generation and its efficacy	PHASE1	10
NCT04011059	Randomized Study of Coronary Revascularization with Intramyocardial Injection of Wharton’s Jelly-derived Mesenchymal Stem Cells	scorem-cells	UNKNOWN	Cardiovascular Diseases, Heart Failure, Coronary Artery Disease	Wharton’s jelly-derived mesenchymal stem cells	Heart Failure	Intramyocardial injection	Left ventricular ejection fraction, Percentage of scar tissue	Estimated functional status, Recovery of the ejection fraction	PHASE1/PHASE2	40
NCT04396899	Safety and Efficacy of Induced Pluripotent Stem Cell-derived Engineered Heart Muscle for Advanced Heart Failure	BioVAT-HF	RECRUITING	Heart Failure	Engineered heat muscle	Severe Heart Failure	Intramyocardial injection	Target heart wall thickness, Target heart wall motion	NaN	PHASE1/PHASE2	53

Unlike many preclinical studies that use animal models of acute MI, patients who are targeted in clinical trials consist mainly of those with chronic heart failure. In cases of severe heart failure, extensive tissue damage is present with irreversible remodeling and fibrosis. For these patients, tissue transplantation is considered necessary for curative treatment, and further research into the creation of fully vascularized tissue grafts capable of long-term survival post-transplantation is of utmost importance.

## 4. Discussion

Cardiac tissue regeneration remains a significant challenge, primarily due to the limited regenerative potential of heart tissue. While ECM patches can provide mechanical support to damaged myocardium, the stimulation of in situ regeneration remains difficult to achieve [13]. Even with the administration of growth factors in conjunction with ECM patches, the effects on cardiac regeneration are limited. Moreover, while vasculogenesis induced by such growth factors can be beneficial in treating acute MI, the efficacy of this approach is significantly reduced in cases of chronic heart damage [14,50,117]. This highlights the importance of creating functional artificial tissues for heart regeneration through tissue engineering.

Adequate vascularization is essential for the survival of transplanted cells and the regeneration of functional myocardium. Currently, the implantation of functional and sufficiently sized tissue constructs has been limited by poor survival, especially in large animal models, with only microvasculature for blood supply [23,112,156]. Therefore, attempts are being made to form tissues with feeding arteries and drainage veins that can increase perfusion. In vitro studies have shown that the use of contemporary 3D printer technologies can improve cell survival rates by creating pre-formed vascular structures. It is now possible to print capillary-sized blood vessels, and the reproduction of tissue perfusion in an in vitro environment is becoming highly sophisticated [26,89]. However, regardless of the success of in vitro models, further research is needed to produce in vivo models and pave the way for the clinical application of bioengineered tissue grafts. Moreover, methods to improve the survival and integration of implanted tissues, ensure functional maturation of engineered myocardial tissues, and address the host immune response must be developed before heart regeneration may become a viable therapeutic option.

### 4.1. Functional Integration

In a study by Jabbour et al., large engineered cardiac tissue patches consisting of hiPSC-CMs embedded in a fibrin-based hydrogel were implanted over the epicardium of infarcted rabbit hearts. Results showed that implantation of these patches was associated with improved cardiac function, reduced infarct size, and enhanced vascularization, without incidences of arrhythmia. However, electrical coupling between the patch and host myocardium was not observed [157]. It may be inferred that while the pericardial patch promotes tissue regeneration within the area of infarction, it does not functionally link with the host myocardium. Other studies have reported electrical activity in cell sheet implants, but achieving full electromechanical integration remains a significant challenge. Komae et al. found that while their multi-layered cell sheets showed electrical activity and pulsatile function, the primary benefit was often paracrine effects rather than full integration [24]. Ultimately, it may be more desirable to use approaches such as interposition grafts or the intramyocardial injection of cells, in which the transplanted tissue directly interfaces with the host myocardium without an intervening epicardium. On the other hand, graft-initiated host excitation, or engraftment arrhythmias arising from positive graft–host electrical coupling, are a potential complication that necessitates close monitoring following cardiac tissue transplantation in vivo [158,159].

### 4.2. Host Immune Reaction

**Figure 3:**
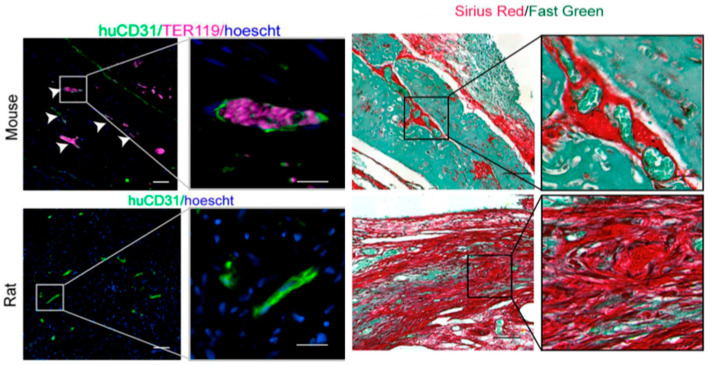
Differences in vascular formation and fibrosis after cardiac patch transplantation in thymectomized mice and rats. In thymectomized mice, a well-established vascular structure with mouse blood components (TER119) within the vessels is observed (White arrows indicate vascular cord). Additionally, Sirius red staining indicates relatively mild fibrosis in the transplanted cardiac patch. Conversely, similar experiments in thymectomized rats show poor vascular development and significant fibrosis in the transplanted patch. Adapted from ref. [115].

The host immune response to transplanted tissues has a significant effect on successful engraftment and long-term survival. Host reactions to the introduction of human cells may vary depending on the species. Establishing appropriate immunosuppressive models is needed to advance these therapies. Brady et al. demonstrated that while athymic mice were capable of supporting chimeric vasculature, athymic rats exhibited a significant inflammatory response and collagen deposition, resulting in less established vasculature [115] (Figure 3). Long-term engraftment in either species was not assessed in this study. Nevertheless, these findings highlight the importance of selecting a suitable animal model for the preclinical testing of engineered tissues. A study by Itoh et al. reported the development of an immunodeficient swine model, which allowed for the long-term accommodation of artificial human vascular tubes [160].

Currently, there is no well-established immunosuppressive regimen for large animals receiving xenogeneic iPSC cells and their derivatives. A study by Ito et al. endeavored to characterize the long-term rate of survival for hiPSC-CM cardiac patches implanted in rats based on the duration of immunosuppressive treatment [161]. They found that even with long-term immunosuppression, the cardiac patch eventually disappeared. Furthermore, no differences in cardiac function, inhibition of fibrosis, or capillary density were observed as a result of differences in the duration of immunosuppression. Nevertheless, with xenotransplantation of human cells, sufficient immunosuppressive effects may not be achievable with medication alone. Thus, the use of allogeneic cells in preclinical studies may represent the most desirable method for evaluating the therapeutic effect of tissue transplantation.

While the field of cardiac regenerative medicine has garnered significant interest, challenges such as tissue vascularization, functional integration with host tissue after transplantation, and differences in the response of animal models to transplanted tissues must be resolved before cardiac tissue engineering can achieve sufficient clinical efficacy. Addressing these issues will likely require further research into each specific problem. However, overcoming these challenges may ultimately pave the way for the realization of whole-heart creation and organ transplantation.

## 5. Conclusions

The field of cardiac regeneration has evolved significantly within the past two decades. The ability to engineer cardiac tissues with pre-formed macrovasculature, as well as to induce the formation of microvasculature, shows remarkable technological advancement. The ability to create tissues that can support circulation has been demonstrated in in vitro studies. However, these techniques have not yet been applied in an in vivo setting. Vascularization, host immunogenicity, and functional integration remain significant challenges to the successful clinical application of engineered tissue transplantation.

## Data Availability

Data are available if requested.
